# Differential diagnosis between NMOSD and MS: a retrospective study based on clinical and imaging features

**DOI:** 10.3389/fneur.2026.1718736

**Published:** 2026-01-20

**Authors:** Ya-Nan Ma, Qian Wang, Si-Rong Ma, Li Yang, Yong Ding, Qing-Qiu Wu

**Affiliations:** 1 Department of Geriatrics and Specialty Medicine, General Hospital of Ningxia Medical University, Yinchuan, Ningxia, China; 2 Department of Neurophysiology, Ningbo Medical Center Li Huili Hospital, Ningbo, Zhejiang, China; 3 School of Basic Medical Sciences, Ningxia Medical University, Yinchuan, Ningxia, China; 4 Emergency Department, General Hospital of Ningxia Medical University, Yinchuan, Ningxia, China

**Keywords:** clinical features, differential diagnosis, MS, NMOSD, prognosis

## Abstract

**Background:**

Neuromyelitis optica spectrum disorders (NMOSD) and multiple sclerosis (MS) are both inflammatory demyelinating diseases of the central nervous system, sharing many similarities in clinical manifestations. However, they differ significantly in terms of etiology, pathological mechanisms, treatment, and prognosis. Since early clinical differentiation can be challenging, achieving an accurate diagnosis at the initial stage of disease onset is particularly critical. Therefore, a thorough analysis of patients’ clinical characteristics is of great importance in assisting clinicians with early diagnosis and treatment, ultimately contributing to improved patient outcomes.

**Methods:**

Clinical data were collected for patients who were first diagnosed with NMOSD or MS at the General Hospital and the Cardiovascular and Cerebrovascular Hospital of Ningxia Medical University between January 2018 and January 2022. Collected information included demographic data, past medical history, initial clinical symptoms, physical examination findings, laboratory tests, imaging studies, and three types of evoked potentials. Patients were followed up for relapse during remission, presence of residual symptoms, medication use, and scores on the Extended Disability Status Scale (EDSS). The clinical characteristics of the two diseases were summarized and the results subjected to statistical analysis.

**Results:**

This study conducted a comparative analysis across multiple indicators, comprehensively revealing significant differences in the clinical characteristics of NMOSD and MS. The results showed that the proportion of female patients was significantly higher in the NMOSD group (86.2%) compared with the MS group (69.0%), with a statistically significant difference (*p* = 0.035). In terms of clinical manifestations, NMOSD patients more frequently presented with comorbid autoimmune diseases, initial symptoms, and neurological signs at admission, all of which were more severe and common than in MS patients, with statistically significant differences. The severity of neurological dysfunction in NMOSD patients during the acute phase was also markedly greater than that observed in MS patients, as confirmed by comparisons of the EDSS scores. Laboratory examinations further demonstrated fundamental differences between NMOSD and MS in cerebrospinal fluid characteristics, specific antibodies, and other serological markers, providing important evidence for differential diagnosis. In addition, imaging and electrophysiological findings indicated that MS lesions were predominantly located in the brain, whereas NMOSD lesions mainly involved the optic nerve and spinal cord. Notably, NMOSD patients exhibited more extensive spinal cord involvement and more frequent impairment of the visual pathway.

**Conclusion:**

Although NMOSD and MS share many similarities in clinical symptoms, they differ substantially in their fundamental characteristics, as reflected in demographic features, clinical manifestations, laboratory and imaging findings, as well as prognosis. Compared with MS, NMOSD patients are typically older at disease onset, have a higher proportion of females, and experience more frequent relapses and greater disability. In terms of imaging, MS lesions are predominantly distributed in the brain, whereas NMOSD mainly involves the optic nerve and spinal cord. Therefore, early differentiation between the two diseases in clinical practice is essential for developing targeted treatment strategies and ultimately improving patient outcomes.

## 1 Introduction

Neuromyelitis optica spectrum disorders (NMOSD) evolved from the concept of neuromyelitis optica (NMO) and represent a refined disease entity. As an inflammatory demyelinating disorder of the central nervous system (CNS), NMOSD is primarily characterized by involvement of the optic nerve and spinal cord. In its early stage, the disease was once considered a subtype of multiple sclerosis (MS) (1, 2). In 2004, Lennon and colleagues identified neuromyelitis optica immunoglobulin G (NMO-IgG) in the serum of some NMO patients, which specifically binds to aquaporin-4 (AQP4) located on the endfeet of astrocytes. This antibody was later designated as AQP4-IgG. The key significance of this discovery lies in the fact that AQP4-IgG is typically positive in NMO patients but negative in MS patients, thereby providing crucial serological evidence that led to the reclassification of NMO as a disease distinct from MS (3, 4). Serum positivity for AQP4-IgG in NMO demonstrates both high specificity and high sensitivity. Owing to the strong specificity of this biomarker, the revised 2006 diagnostic criteria for NMO formally incorporated AQP4-IgG as a core evaluation indicator, while also relaxing the absolute restriction that CNS lesions be confined solely to the optic nerve and spinal cord (5). With growing recognition of the diagnostic complexity and disease heterogeneity in patients who are seronegative for AQP4-IgG, the International Panel for NMO Diagnosis updated the diagnostic criteria for NMOSD in 2015. Under the revised criteria, NMOSD was classified into three categories based on serum AQP4-IgG status: positive, negative, or unknown (6).

NMOSD is a relatively rare inflammatory demyelinating disease of the central nervous system, occurring predominantly in young and middle-aged adults, though it can also affect both the elderly and children, with a clear predominance among female patients (1, 2, 7, 8). NMOSD often has an acute onset and is characterized by a high relapse rate, with frequent and severe relapses leading to progressive neurological dysfunction. Without timely and effective treatment, patients may develop serious and persistent impairments in visual and motor functions, such as vision loss or even blindness, limb weakness, numbness, paralysis, and bladder or bowel dysfunction. These deficits not only severely affect daily living and quality of life but also impose substantial psychological and financial burdens on families (2, 9). Current treatments for the acute phase of NMOSD primarily include corticosteroids, plasma exchange (PE), and intravenous immunoglobulin (IVIG), with the main goals of alleviating acute clinical symptoms, shortening the duration of attacks, and promoting early recovery of neurological function. However, most patients experience relapses within a few years. Therefore, once a diagnosis is confirmed, long-term maintenance therapy should be initiated promptly. The choice of medications during the remission phase is crucial, as selecting appropriate immunosuppressive agents based on disease severity can reduce relapse rates and delay the accumulation of disability (10–14).

MS is a chronic inflammatory disease of the CNS, characterized pathologically by demyelination and axonal degeneration, with considerable heterogeneity in clinical manifestations, disease course, and prognosis. Its pathogenesis primarily involves autoreactive T helper 1 (Th1) and Th17 cells. Upon activation by an unknown antigen, Th1 cells first produce proinflammatory cytokines, while Th17 cells secrete IL-17. These cytokines subsequently upregulate the expression of specific matrix metalloproteinases, disrupt the blood–brain barrier, and ultimately mediate T-cell infiltration into the central nervous system (15–17). MS primarily affects young and middle-aged adults, with a significantly higher incidence in females than males, although the female predominance is slightly lower than that observed in NMOSD (18). Studies have confirmed that preceding infections are one of the important triggers for MS. Lesions can simultaneously affect multiple regions of the CNS, and the acute phase often presents with a variety of neurological symptoms. Many patients experience delayed diagnosis and treatment, resulting in poor prognosis and a significant impact on quality of life (19).

Both NMOSD and MS are autoimmune demyelinating diseases of the CNS, and the involvement of specific anatomical sites can lead to similar clinical symptoms and manifestations in patients. However, the treatment approaches for the two diseases differ markedly. For example, medications such as interferons are effective in treating MS but are ineffective in NMOSD and may even exacerbate disease progression, leading to clinical deterioration (20–22). Such therapeutic differences also exist in other MS treatment regimens. Notably, a study has confirmed that AQP4 antibody-positive NMOSD patients misdiagnosed with MS and treated with natalizumab fail to achieve effective control of disease activity, with a significantly increased relapse rate (23). This risk was also observed with fingolimod therapy, the first orally administered medication approved in the United States for relapsing–remitting MS (24, 25). Related case reports have also revealed that NMOSD patients experienced severe outcomes such as worsening brain lesions during treatment with fingolimod (26, 27). Therefore, early, rapid, and accurate diagnosis, combined with appropriate treatment, is particularly crucial for reducing the risk of relapse and mortality, as well as for improving disability outcomes.

This study collected demographic data, medical history, physical examination findings, clinical manifestations, and relevant ancillary test results from patients who were first diagnosed with NMOSD or MS. Follow-up was conducted to assess relapse during remission, residual symptoms, medication use, and scores on the Expanded Disability Status Scale (EDSS). By systematically comparing the similarities and differences between the two diseases, the study aims to provide a basis for early diagnosis, differential diagnosis, and prognosis assessment, enhance clinicians’ understanding of both conditions, reduce misdiagnosis and missed diagnosis, and ultimately improve patient outcomes and quality of life.

## 2 Materials and methods

### 2.1 Study subjects

The study subjects were patients who were first diagnosed with NMOSD or MS and admitted to the General Hospital of Ningxia Medical University and the Cardiovascular and Cerebrovascular Hospital of Ningxia Medical University between January 2018 and January 2022.

### 2.2 Inclusion criteria

(1) All NMOSD patients met the 2015 International Consensus Diagnostic Criteria for NMOSD (6) (Appendix 1).
(2) All MS patients met the 2017 revised McDonald diagnostic criteria (28) (Appendix 2).
(3) First diagnosis of NMOSD or MS.
(4) Relatively complete acute-phase data, including lumbar puncture and relevant imaging examinations.

### 2.3 Exclusion criteria

(1) Other CNS diseases that may interfere with diagnosis or disease assessment, such as acute disseminated encephalomyelitis, autoimmune encephalitis, primary or metastatic tumors of the nervous system.
(2) Motor system dysfunction caused by other diseases that prevented cooperation with examinations.
(3) Patients who had a confirmed diagnosis of other immunodeficiency diseases prior to admission or who had received immunosuppressive therapy before admission.
(4) Failure of vital organs.

## 3 Methods

### 3.1 Study design

Clinical data of patients with a first diagnosis of NMOSD or MS admitted to the two hospitals between January 2018 and January 2022 were retrospectively collected from the electronic medical record system. Differences between the two groups were compared. After acute-phase treatment and discharge, patients were followed up in the outpatient clinic, with a minimum follow-up period of 1 year.

### 3.2 Data collection

#### 3.2.1 Demographic data

Sex and age at first symptom onset were collected.

#### 3.2.2 Clinical data

The following clinical information was collected:

(1) Basic characteristics: mode of onset, defined as:
• Acute onset: symptoms peaked within 1 week after onset.
• Subacute onset: symptoms peaked within 1 week to 1 month.
• Chronic onset: symptoms peaked more than 1 month after onset.
(2) Season of onset, time to diagnosis, disease duration, initial precipitating factors, first department visited, and comorbidities.
(3) Clinical symptoms and assessment: first symptoms, detailed neurological physical examination results, and Expanded Disability Status Scale (EDSS) score.
(4) Auxiliary examinations:
• Laboratory tests: serum AQP4-IgG, MOG-IgG, and other specific antibody tests (according to available data).
• Imaging: cranial and spinal magnetic resonance imaging (MRI) characteristics and reports.
• Evoked potentials: visual evoked potential (VEP), brainstem auditory evoked potential (BAEP), and somatosensory evoked potential (SEP).

#### 3.2.3 Follow-up and outcome measures

All patients were followed up by telephone or outpatient visits during the remission period. Data collected included relapse within 1 year after discharge, EDSS score at 1 year after discharge, presence of residual symptoms, and medications.

Relapse was defined as a ≥ 4-week interval between the first and second attacks, with the second attack meeting the diagnostic criteria for NMOSD or MS (6).

#### 3.2.4 EDSS

The EDSS scoring system is based on eight functional systems of the CNS (visual, brainstem, cerebellar, cerebral, motor, sensory, ambulation, bladder/rectum), each divided into 5–6 grades. The complete EDSS score ranges from 0 to 10, with higher scores indicating more severe neurological dysfunction (29). The scoring form is provided in Appendix 3.

#### 3.2.5 Laboratory data

The cerebrospinal fluid (CSF) pressure and laboratory results obtained during hospitalization were collected, along with serum and/or CSF AQP4 antibody, oligoclonal band (OCB), and non–organ-specific autoantibody test results. Serum and/or CSF AQP4-IgG was detected using a cell-based assay (CBA) with HEK293 cells transfected with the human AQP4-M23 isoform as the antigen substrate, strictly following the manufacturer’s instructions.

#### 3.2.6 Imaging data

All patients underwent MRI examinations using 1.5 T scanner with standardized acquisition protocols. The MRI sequences included T1-weighted, T2-weighted, and fluid-attenuated inversion recovery (FLAIR) images for the brain, and sagittal and axial T2-weighted images for the spinal cord. Lesion locations in the brain and spinal cord were recorded, and the length of spinal cord lesions was expressed as the number of vertebral segments involved. In cases with multiple lesions, the longest lesion segment was used for analysis. Imaging data were independently reviewed by two experienced neuroradiologists.

### 3.3 Study groups

According to the inclusion and exclusion criteria, a total of 123 patients were enrolled, including 94 NMOSD patients and 29 MS patients.

### 3.4 Statistical analysis

Data were analyzed using SPSS version 26.0. Continuous variables with a normal distribution were expressed as mean ± standard deviation and compared using the independent-samples *t*-test. Non-normally distributed data were expressed as median (interquartile range) and compared using the Mann–Whitney U test. Categorical variables were expressed as frequency (%) and analyzed using the chi-square test or Fisher’s exact test. A *p*-value < 0.05 was considered statistically significant.

## 4 Results

### 4.1 Comparison of general characteristics between NMOSD and MS patients

A total of 123 patients were included in this study, comprising 94 cases of NMOSD and 29 cases of MS. Among NMOSD patients, 81 (85.11%) were female, with a mean age of 45.61 ± 15.69 years; among MS patients, 20 (68.97%) were female, with a mean age of 39.62 ± 14.91 years. NMOSD patients presented with a later age of onset and a higher female predominance, with a statistically significant difference in sex distribution between the two groups (*p* = 0.035). The median time from initial symptoms to confirmed diagnosis was 31.00 (17.00, 365.00) days in the NMOSD group and 180.00 (27.00, 912.50) days in the MS group. The median disease duration was 31.00 (18.75, 40.00) months for NMOSD and 54.00 (30.00, 62.50) months for MS. The differences in diagnostic delay and disease duration between the two groups were statistically significant (*p* < 0.05). No significant differences were found in terms of disease onset season or initial disease pattern (*p* > 0.05). Chi-square test was used for sex, onset season, and initial disease pattern; independent-sample *t*-test was used for age at onset; Mann–Whitney U test was applied for diagnostic delay and disease duration (Table 1).

**Table 1:** Comparison of general characteristics between patients with NMOSD and MS.

Characteristics	NMOSD (*n* = 94)	MS (*n* = 29)	*P*
Female (%)	81 (86.17)	20 (68.97)	0.035^*^
Age at onset (years)	45.61 ± 15.69	39.62 ± 14.91	0.072
Season of onset, *n* (%)			
Spring	22 (23.40)	6 (20.69)	0.761
Summer	20 (21.28)	9 (31.03)	0.279
Autumn	27 (28.72)	8 (27.59)	0.906
Winter	25 (26.60)	6 (20.69)	0.522
Mode of onset, *n* (%)			
Acute onset	28 (29.79)	10 (34.48)	0.632
Subacute onset	40 (42.55)	11 (37.93)	0.659
Chronic onset	26 (27.66)	8 (27.59)	0.994
Time to diagnosis (days)	31 (17.00, 365.00)	180 (27.00, 912.50)	0.039^*^
Disease duration (months)	31 (18.75, 40.00)	54 (30.00, 62.50)	<0.001^*^

NMOSD, neuromyelitis optica spectrum disorder; MS, multiple sclerosis. * Denotes *p* < 0.05.

### 4.2 Comparison of initial onset triggers between NMOSD and MS patients

Regarding initial onset triggers, 36 NMOSD patients (38.29%) and 10 MS patients (34.48%) had identifiable triggers. In both groups, antecedent infections were the most common. Seventeen NMOSD patients (18.09%) experienced antecedent infections, including upper respiratory tract infection (*n* = 7), pulmonary infection (*n* = 4), herpes zoster virus infection (*n* = 3), and urinary tract infection (*n* = 3). Three MS patients (10.34%) had antecedent infections, including upper respiratory tract infection (*n* = 2) and gastrointestinal infection (*n* = 1). No statistically significant differences were observed between the two groups in terms of the presence or type of triggers (*p* > 0.05). Chi-square test was used for the presence of onset triggers, while Fisher’s exact test was applied for other specific comparisons (Table 2).

**Table 2:** Comparison of precipitating factors for the first onset between NMOSD and MS patients.

Precipitating factors	NMOSD (*n* = 94)	MS (*n* = 29)	*P*
With identifiable precipitating factors, *n* (%)	36 (38.29)	10 (34.48)	0.710
Preceding infection, *n* (%)	17 (18.09)	3 (10.34)	0.401
Fatigue, *n* (%)	4 (4.26)	2 (6.8)	0.625
Pregnancy/Postpartum, *n* (%)	3 (3.19)	0 (0.00)	1.000
Vaccination, *n* (%)	2 (2.13)	1 (3.45)	0.557
Trauma, *n* (%)	1 (1.06)	0 (0.00)	1.000
Chill exposure, *n* (%)	2 (2.13)	1 (3.45)	0.557
Neuropsychiatric symptoms, *n* (%)	1 (1.06)	0 (0.00)	1.000
Binge eating, *n* (%)	2 (2.13)	0 (0.00)	1.000
Stress-related onset, *n* (%)	4 (4.26)	2 (6.90)	0.625
Onset after grief, *n* (%)	0 (0.00)	1 (3.45)	0.236

### 4.3 Comparison of initial departments visited by NMOSD and MS patients

Due to the diversity of initial symptoms, the departments first visited by NMOSD and MS patients varied. The most frequently visited departments were neurology and ophthalmology in both groups. In the NMOSD group, 32 patients (34.04%) first visited neurology, 20 (21.28%) visited ophthalmology, and 42 (44.68%) visited other departments. In the MS group, 15 patients (51.72%) first visited neurology, 7 (24.14%) visited ophthalmology, and 7 (24.14%) visited other departments. There was no statistically significant difference between the two groups in terms of the first department visited (*p* > 0.05). Chi-square test was used for neurology and ophthalmology visits, while Fisher’s exact test was used for other departments (Table 3).

**Table 3:** Comparison of initial medical consultation departments between NMOSD and MS patients.

Department of first medical contact	NMOSD (*n* = 94)	MS (*n* = 29)	*P*
Department of Neurology, *n* (%)	32 (34.04)	15 (51.72)	0.087
Department of Ophthalmology, *n* (%)	20 (21.28)	7 (24.14)	0.745
Department of Gastroenterology, *n* (%)	8 (8.51)	0 (0.00)	0.196
Emergency Department, *n* (%)	14 (14.89)	2 (6.90)	0.355
Department of Traditional Chinese Medicine, *n* (%)	11 (11.70)	1 (3.45)	0.291
Department of Neurosurgery, *n* (%)	4 (4.17)	1 (3.45)	1.000
Department of Orthopedics, *n* (%)	4 (4.17)	3 (10.34)	0.354
Department of Rheumatology, *n* (%)	1 (1.06)	0 (0.00)	1.000

### 4.4 Comparison of comorbidities between NMOSD and MS patients

With regard to past surgical history, 25 NMOSD patients (34.04%) had undergone surgery, including cesarean section (*n* = 7), cholecystectomy (*n* = 5), thyroidectomy (*n* = 3), breast fibroadenoma resection (*n* = 2), hip surgery (*n* = 2), appendectomy (*n* = 2), inguinal hernia repair (*n* = 1), salpingectomy (*n* = 1), adrenal tumor resection (*n* = 1), and cervical cancer surgery (*n* = 1). In contrast, 4 MS patients (13.79%) had surgical histories, including cholecystectomy (*n* = 2), inguinal hernia repair (*n* = 1), and cardiac radiofrequency ablation (*n* = 1). The difference in surgical history was not statistically significant (*p* > 0.05). In terms of autoimmune comorbidities, 19 NMOSD patients (20.21%) had concomitant autoimmune diseases, including autoimmune thyroid disease (*n* = 6), Sjögren’s syndrome (*n* = 5), systemic lupus erythematosus (*n* = 3), rheumatoid arthritis (*n* = 1), undifferentiated connective tissue disease (*n* = 2), and scleroderma (*n* = 1). Only 1 MS patient (3.45%) had an autoimmune disease (rheumatoid arthritis). The incidence of autoimmune comorbidities was significantly higher in NMOSD (*p* = 0.042). No significant differences were found in other comorbidities (*p* > 0.05). Chi-square test was applied for surgical history, and Fisher’s exact test for other comorbidities (Table 4).

**Table 4:** Comparison of comorbidities between NMOSD and MS patients.

Comorbidities	NMOSD (*n* = 94)	MS (*n* = 29)	*P*
History of prior surgery, *n* (%)	25 (34.04)	4 (13.79)	0.156
Hypertension, *n* (%)	15 (15.96)	6 (20.69)	0.577
Diabetes mellitus, *n* (%)	5 (5.15)	2 (6.90)	0.668
Coronary heart disease, *n* (%)	4 (4.26)	2 (6.90)	0.625
Congenital heart disease, *n* (%)	3 (3.19)	0 (0.00)	1.000
Cardiac arrhythmia, *n* (%)	5 (5.15)	2 (6.90)	0.668
Hyperlipidemia, *n* (%)	10 (10.64)	4 (13.79)	0.739
Fatty liver disease, *n* (%)	5 (5.15)	1 (3.45)	1.000
Autoimmune diseases, *n* (%)	19 (20.21)	1 (3.45)	0.042^*^
Chronic pulmonary diseases, *n* (%)	3 (3.19)	2 (6.90)	0.337
Epilepsy, *n* (%)	2 (2.13)	0 (0.00)	1.000
Neoplasms, *n* (%)	2 (2.13)	0 (0.00)	1.000
Psychiatric disorders, *n* (%)	9 (9.57)	1 (3.45)	0.449
Anemia, *n* (%)	12 (12.77)	1 (3.45)	0.186
Cerebral infarction, *n* (%)	6 (6.38)	2 (6.90)	1.000
Others, *n* (%)	4 (4.26)	3 (10.34)	0.354

* Denotes *p* < 0.05. A single patient may have multiple comorbid conditions.

### 4.5 Comparison of initial clinical manifestations between NMOSD and MS patients

In terms of initial symptoms, NMOSD patients were more likely than MS patients to present with sensory disturbances (77.17% vs. 55.17%, *p* = 0.035), motor dysfunction (62.77% vs. 41.37%, *p* = 0.042), visual impairment (44.68% vs. 24.13%, *p* = 0.048), and bladder/rectal dysfunction (46.81% vs. 10.34%, *p* < 0.001), all with statistically significant differences. No significant differences were found between the two groups in other initial symptoms (*p* > 0.05). Chi-square test was used for sensory disturbances, motor dysfunction, visual impairment, dizziness, and gait instability, while Fisher’s exact test was applied for other symptoms (Table 5).

**Table 5:** Comparison of initial symptoms between NMOSD and MS patients.

Initial symptoms	NMOSD (*n* = 94)	MS (*n* = 29)	*P*
Sensory disturbances, *n* (%)	71 (77.17)	16 (55.17)	0.035^*^
Motor dysfunction, *n* (%)	59 (62.77)	12 (41.37)	0.042^*^
Visual impairment, *n* (%)	42 (44.68)	7 (24.13)	0.048^*^
Bladder-bowel dysfunction, *n* (%)	44 (46.81)	3 (10.34)	0.000^*^
Dizziness, *n* (%)	24 (29.79)	7 (31.03)	0.880
Nausea and vomiting, *n* (%)	17 (18.09)	1 (3.45)	0.070
Gait instability, *n* (%)	19 (20.21)	4 (13.79)	0.438
Involuntary movements, *n* (%)	8 (8.51)	0 (0.00)	0.196
Intractable hiccups, *n* (%)	5 (5.32)	0 (0.00)	0.591
Ocular/Orbital pain, *n* (%)	4 (4.26)	1 (3.45)	1.000
Nystagmus/Oculomotor dysfunction, *n* (%)	9 (9.57)	4 (13.79)	0.503
Dysarthria, *n* (%)	3 (3.19)	0 (0.00)	1.000
Dysphagia, *n* (%)	2 (2.13)	1 (3.45)	0.557

* Denotes *P* < 0.05. Some patients may present with multiple concurrent clinical symptoms.

### 4.6 Comparison of physical examination findings at admission between NMOSD and MS patients

At admission, 40 NMOSD patients (42.55%) had muscle strength graded 0–3, compared with 4 MS patients (13.79%). The degree of disability was significantly greater in NMOSD patients (*p* < 0.05). No significant differences were observed in other physical examination findings (*p* > 0.05). Fisher’s exact test was applied for hypertonia, hypotonia, and ataxia, while chi-square test was used for other parameters (Table 6).

**Table 6:** Comparison of admission physical examination findings between NMOSD and MS patients.

Physical examination	NMOSD (*n* = 94)	MS (*n* = 29)	*P*
Muscle strength grade 0–3, *n* (%)	40 (42.55)	4 (13.79)	0.005^*^
Sensory level impairment, *n* (%)	36 (38.30)	6 (20.69)	0.080
Hypertonia, *n* (%)	4 (4.26)	3 (10.34)	0.354
Hypotonia, *n* (%)	4 (4.26)	0 (0.00)	0.572
Decreased superficial reflexes, *n* (%)	33 (35.11)	6 (20.69)	0.145
Hyperactive deep tendon reflexes, *n* (%)	52 (55.32)	10 (34.48)	0.050
Decreased deep tendon reflexes, *n* (%)	26 (27.66)	4 (13.79)	0.128
Ataxia, *n* (%)	14 (14.89)	5 (17.24)	0.772

* Denotes *P* < 0.05. Some patients may present with multiple concurrent physical signs.

### 4.7 Comparison of EDSS scores and follow-up outcomes between NMOSD and MS patients

After excluding patients lost to follow-up, refusing follow-up, or deceased by the end of follow-up, 94 patients remained, including 72 NMOSD and 22 MS cases. The median EDSS score at admission during the acute phase was 4.00 (3.00, 6.50) for NMOSD and 2.50 (1.89, 3.50) for MS; the median EDSS score at discharge was 2.50 (1.50, 5.00) for NMOSD and 2.00 (1.00, 2.50) for MS. NMOSD patients had significantly higher EDSS scores during the acute phase (*p* = 0.001 and *p* = 0.023, respectively). No significant differences were observed between the two groups in EDSS scores at 1 year post-discharge, relapse within 1 year, residual symptoms, or use of immunosuppressants (*p* > 0.05). Mann–Whitney U test was used for EDSS scores, chi-square test for relapse and immunosuppressant use, and Fisher’s exact test for residual symptoms (Table 7).

**Table 7:** Comparison of EDSS scores and follow-up data between NMOSD and MS patients.

Characteristics	NMOSD (*n* = 72)	MS (*n* = 22)	*P*
Admission EDSS score	4.00 (3.00, 6.50)	2.50 (1.89, 3.50)	0.001^*^
Discharge EDSS score	2.50 (1.50, 5.00)	2.00 (1.00, 2.50)	0.023^*^
EDSS score at 1 year after discharge	2.50 (1.00, 5.00)	1.00 (1.00, 3.00)	0.053
Relapse within 1 year after discharge, *n* (%)	37 (51.39)	8 (36.36)	0.217
Residual symptoms, *n* (%)	64 (88.89)	16 (72.73)	0.068
Immunosuppressant use, *n* (%)	22 (30.56)	4 (18.18)	0.256

EDSS, expanded disability status scale; * denotes *P* < 0.05.

### 4.8 Comparison of laboratory findings at admission between NMOSD and MS patients

#### 4.8.1 Cerebrospinal fluid

The incidence of elevated CSF leukocytes (57.45% vs. 31.03%, *p* = 0.013) and lymphocytic predominance (55.32% vs. 31.03%, *p* = 0.022) was significantly higher in NMOSD compared with MS. Median CSF protein level was also significantly higher in NMOSD [0.52 (0.37, 0.78) g/L] than in MS [0.44 (0.33, 0.55) g/L, *p* = 0.010]. No significant differences were found in CSF pressure, glucose, or chloride levels (*p* > 0.05). Chi-square test was used for pressure elevation, leukocytosis, and lymphocytic predominance; Mann–Whitney U test was used for protein and glucose levels; independent-sample *t*-test was used for chloride (Table 8).

**Table 8:** Comparison of cerebrospinal fluid findings between NMOSD and MS patients.

Cerebrospinal fluid (CSF)	NMOSD (*n* = 94)	MS (*n* = 29)	*P*
Increased CSF pressure, *n* (%)	10 (10.64)	3 (10.34)	1.000
Elevated white blood cell (WBC) count, *n* (%)	54 (57.45)	9 (31.03)	0.013^*^
Lymphocytic predominance, *n* (%)	52 (55.32)	9 (31.03)	0.022^*^
Protein (g/L)	0.52 (0.37, 0.78)	0.44 (0.33, 0.55)	0.010^*^
Glucose (mmol/L)	3.00 (2.70, 3.50)	2.90 (2.70, 3.55)	0.884
Chloride (mmol/L)	123.03 ± 0.35	123.90 ± 0.43	0.198

* Denotes *P* < 0.05.

#### 4.8.2 AQP4 antibody and oligoclonal bands

Among patients who underwent both AQP4 antibody and OCB testing (*n* = 84 and *n* = 26, respectively), 64 NMOSD patients (76.19%) were AQP4-positive and 3 (11.54%) were OCB-positive, while none of the MS patients (0%) were AQP4-positive and 17 (65.38%) were OCB-positive. These differences were statistically significant (*p* < 0.001). All comparisons were performed using chi-square test (Table 9).

**Table 9:** Comparison of AQP4 antibody and oligoclonal bands between NMOSD and MS patients.

Laboratory findings	NMOSD (*n* = 84)	MS (*n* = 26)	*P*
AQP4 antibody positive, *n* (%)	64 (76.19)	0 (0)	<0.001^*^
Oligoclonal bands (OCB) positive, *n* (%)	11 (13.10)	17 (65.38)	<0.001^*^

AQP4, aquaporin-4; * denotes *P* < 0.05.

### 4.9 Comparison of imaging findings at admission between NMOSD and MS patients

#### 4.9.1 Brain MRI

The rate of abnormal brain MRI findings was significantly lower in NMOSD compared with MS (55.32% vs. 96.55%, *p* < 0.001). MS patients more frequently exhibited lesions in the subcortical region (7.45% vs. 27.59%, *p* = 0.008), centrum semiovale (24.47% vs. 51.72%, *p* = 0.005), and periventricular region (37.84% vs. 75.86%, *p* < 0.001), whereas no significant differences were found in the basal ganglia, thalamus, periventricular third/fourth ventricle, midbrain, pons, medulla, or cerebellum (*p* > 0.05). Chi-square test was used for abnormal MRI, centrum semiovale, and periventricular lesions; Fisher’s exact test was used for other sites (Table 10).

**Table 10:** Comparison of cranial MRI findings between NMOSD and MS patients.

Cranial MRI	NMOSD (*n* = 94)	MS (*n* = 29)	*P*
Abnormal, *n* (%)	52 (55.32)	28 (96.55)	<0.001^*^
Subcortical, *n* (%)	7 (7.45)	8 (27.59)	0.008^*^
Centrum semiovale, *n* (%)	23 (24.47)	15 (51.72)	0.005^*^
Periventricular, *n* (%)	28 (37.84)	22 (75.86)	<0.001^*^
Basal ganglia, *n* (%)	4 (4.26)	2 (6.70)	0.625
Thalamus, *n* (%)	4 (4.26)	1 (3.45)	1.000
Peri-third/fourth ventricle, *n* (%)	5 (5.32)	1 (3.45)	1.000
Midbrain, *n* (%)	7 (7.45)	5 (17.24)	0.152
Pons, *n* (%)	6 (6.38)	4 (13.79)	0.244
Medulla oblongata, *n* (%)	14 (14.89)	4 (13.79)	1.000
Cerebellum, *n* (%)	5 (5.32)	5 (17.24)	0.055

MRI, magnetic resonance imaging; * denotes *P* < 0.05. Some patients may have multiple concurrent lesions.

#### 4.9.2 Spinal cord MRI

**Figure 1:**
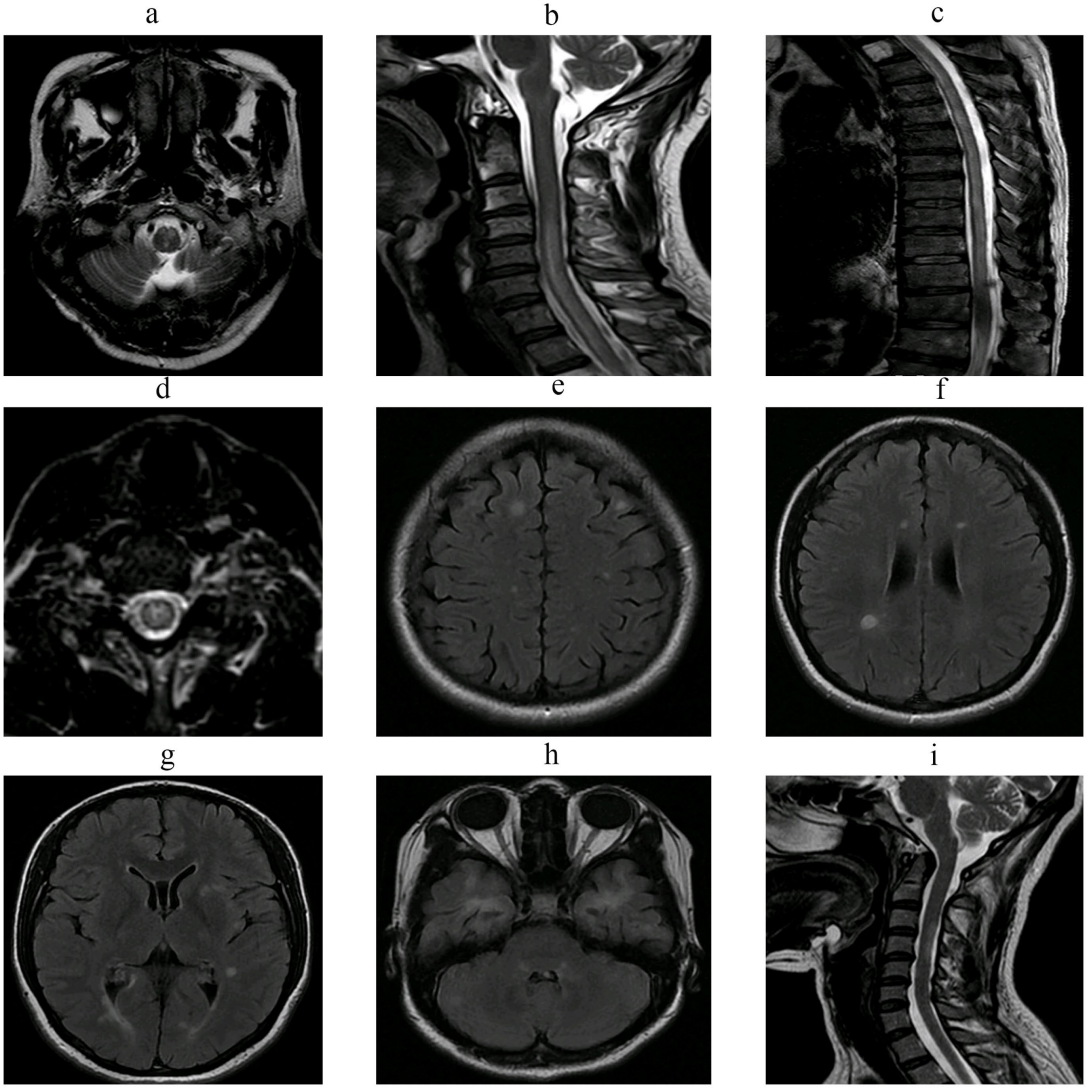
**(a–d)** Show MRI findings of a 48-year-old patient with NMOSD. In **(a)**, patchy abnormal signals are observed in the medulla, presenting as hyperintensity on T2WI. **(b–d)** Demonstrate elongated abnormal intramedullary signals extending from C3 to T11, with the lesion located centrally within the spinal cord on axial view and showing slightly increased signal intensity on T2WI. **(e–i)** Show MRI findings of a 39-year-old patient with MS. **(e–h)** Display multiple ovoid and nodular abnormal signal lesions in the bilateral cerebral hemispheres, right cerebellar hemisphere, and brainstem, appearing as hyperintensity on T2-FLAIR. **(i)** Demonstrates patchy abnormal intramedullary signals in the cervical spinal cord with ill-defined margins, presenting as slightly increased signal intensity on T2WI.

The rate of abnormal spinal cord MRI findings was significantly higher in NMOSD compared with MS (88.30% vs. 58.62%, *p* < 0.001). NMOSD patients more frequently exhibited cervical lesions (88.30% vs. 51.72%, *p* = 0.038), thoracic lesions (67.02% vs. 31.03%, *p* = 0.001), cervicothoracic involvement (53.19% vs. 24.14%, *p* = 0.006), and lesions spanning ≥3 vertebral segments (73.40% vs. 31.03%, *p* < 0.001). Figure 1 illustrates the differences in cranial and spinal MRI findings between NMOSD and MS patients. All comparisons were performed using chi-square test (Table 11).

**Table 11:** Comparison of spinal cord MRI findings between NMOSD and MS patients.

Spinal cord MRI	NMOSD (*n* = 94)	MS (*n* = 29)	*P*
Abnormal, *n* (%)	83 (88.30)	17 (58.62)	<0.001^*^
Affected regions, *n* (%)			
Cervical segment	68 (72.34)	15 (51.72)	0.038^*^
Thoracic segment	63 (67.02)	9 (31.03)	0.001^*^
Cervicothoracic segment	50 (53.19)	7 (24.14)	0.006^*^
≥3 involved segments, *n* (%)	69 (73.40)	9 (31.03)	<0.001^*^

* Denotes *P* < 0.05. Some patients may have multiple concurrent lesions.

#### 4.9.3 Evoked potentials

Evoked potential testing was performed in 81 NMOSD and 24 MS patients. Visual evoked potential (VEP) abnormalities were found in 43 NMOSD patients (53.09%), including 14 with unilateral abnormalities and 29 with bilateral abnormalities, affecting a total of 72 eyes (latency prolongation in 23, amplitude reduction in 11, both latency prolongation and amplitude reduction in 24, and waveform disappearance in 14). In the MS group, 7 patients (29.17%) showed VEP abnormalities, including 2 unilateral and 5 bilateral cases, affecting 12 eyes (latency prolongation in 7, amplitude reduction in 3, both latency prolongation and amplitude reduction in 2). The difference in VEP abnormalities was statistically significant (*p* = 0.039). No significant differences were observed in BAEP or SEP abnormalities (*p* > 0.05). All comparisons were conducted using chi-square test (Table 12).

**Table 12:** Comparison of three evoked potentials between NMOSD and MS patients.

Abnormal evoked potentials	NMOSD (*n* = 81)	MS (*n* = 24)	*P*
VEP (%)	43 (53.09)	7 (29.17)	0.039^*^
BAEP (%)	23 (28.40)	7 (29.17)	0.941
SEP (%)	55 (67.90)	15 (62.50)	0.622

VEP, visual evoked potential; BAEP, brainstem auditory evoked potential; SEP, somatosensory evoked potential; * denotes *P* < 0.05. Some patients may have multiple types of evoked potential abnormalities.

## 5 Discussion

Numerous studies have shown that NMOSD and MS exhibit significant differences in geographic and ethnic distribution, with variations in prevalence, relapse rates, and prognosis across different populations (30–33). Based on a comparison of clinical data, this study further elucidated the similarities and differences between the two diseases across multiple aspects. In terms of demographic characteristics, the proportion of female patients in the NMOSD group was significantly higher than that in the MS group (86.17% vs. 68.97%, *p* = 0.035), consistent with previous epidemiological studies (18, 34, 35). However, there was no significant difference in the age at first onset between the two groups, which may be related to the limited sample size of this study. Regarding disease course, the median time from onset to diagnosis in the NMOSD group was significantly shorter than that in the MS group (31 days vs. 180 days, *p* = 0.039). This difference is likely attributable to the clinical application of AQP4 antibodies as highly specific and sensitive biomarkers for NMOSD, which greatly enhances diagnostic efficiency and reduces misdiagnosis and delayed diagnosis (4). In contrast, the highly heterogeneous clinical manifestations of MS continue to pose challenges for diagnosis (15). Additionally, this study found that the median disease duration in the NMOSD group was significantly shorter than that in the MS group (31 months vs. 54 months, *p* < 0.001). Interpretation of this result should be approached cautiously, taking into account potential factors such as survival bias, enrollment criteria, or differences in disease stage among the included patients. Furthermore, no significant differences were observed between the two groups in terms of onset pattern or seasonal distribution of disease onset.

Beyond the natural course of the disease, precipitating factors are also an important aspect for exploring differences between NMOSD and MS. This study found no significant difference in the overall proportion of patients reporting potential triggers prior to the first onset between the NMOSD and MS groups (38.29% vs. 34.48%, *p* = 0.710), with the most common preceding infections also showing no statistical difference (18.09% vs. 10.34%, *p* = 0.401). This negative result may be related to the limited sample size of the study. It is widely recognized that both NMOSD and MS are influenced by multifactorial etiologies, with infections being one important environmental factor, particularly involving the respiratory, gastrointestinal, or urinary tracts. Epstein–Barr virus (EBV) has long been considered a key environmental factor in MS, while in NMOSD, various pathogens have been reported, including human cytomegalovirus and varicella-zoster virus (36–39). A serological study showed that MS patients exhibited elevated antibody titers against the EBV nuclear antigen, indicating prior infection. In contrast, NMOSD patients demonstrated higher positivity rates and titers for antibodies against the EBV early antigen, suggesting that ongoing viral replication may exacerbate disease progression (40). Furthermore, recent studies have shown that, compared with healthy controls, AQP4-positive NMOSD patients exhibit a significantly higher rate of *Helicobacter pylori* (Hp) infection. Hp may promote neuroinflammatory damage by inducing mast cell degranulation, stimulating a robust Th17 response, and recruiting neutrophils and monocytes (41). Although some studies suggest that the seropositivity rate of Hp is lower in MS patients than in controls, implying a potential protective effect, the association between Hp and MS remains controversial (42, 43).

Both NMOSD and MS predominantly affect women of childbearing age, and immune and hormonal changes during pregnancy are important factors influencing disease activity. Pregnancy-induced immune responses shift from a Th1- to a Th2-dominant profile, which may disrupt immune tolerance and trigger disease relapse (44, 45). The underlying mechanisms differ between the two diseases: in NMOSD, AQP4 expressed in the syncytiotrophoblast of the placenta can serve as a target for autoantibody-mediated attack, with complement activation leading to placental damage. This explains the increased risk of miscarriage and preeclampsia observed in AQP4-positive patients (46, 47). In contrast, the relapse rate of MS decreases during late pregnancy, which is largely attributed to the immunomodulatory effects of elevated estrogen and progesterone levels; however, the postpartum decline in these hormones leads to a rebound in relapse risk (48). Consistent with the findings of this study, three cases (3.19%) of pregnancy- or postpartum-related relapse were reported in the NMOSD group, whereas none were observed in the MS group. Although this trend did not reach statistical significance, it aligns with the notion that NMOSD involves direct placental-targeted mechanisms, while MS is more influenced by hormonal fluctuations, highlighting the unique risks faced by NMOSD patients during pregnancy.

Compared with precipitating factors, comorbidities exhibited more pronounced intergroup differences. With advancing research, comorbidity has become an increasingly recognized clinical concern. NMOSD is often associated with other autoimmune diseases, such as Sjögren’s syndrome (SS), systemic lupus erythematosus (SLE), and rheumatoid arthritis (RA) (49–51). In this study, 19 NMOSD patients (20.21%) had comorbid autoimmune diseases, including SS, SLE, and autoimmune thyroid disease (AITD), which was significantly higher than in the MS group (3.45%, *p* = 0.042). The neurological manifestations of NMOSD and SS often overlap, posing challenges for differential diagnosis. Given that specific anti-AQP4 antibodies can be detected in SS patients with concomitant myelitis or optic neuritis, testing for this antibody has become a highly valuable objective tool for distinguishing coexisting NMOSD from primary neurological involvement of SS (52). In this study, only one MS patient was diagnosed with RA, which may be attributable to the relatively small sample size.

NMOSD and MS are both idiopathic inflammatory demyelinating diseases of the central nervous system, with overlapping clinical manifestations. Many patients with NMOSD and MS experience sensory symptoms, particularly pain (53–55). This study showed that sensory disturbances were the most common initial symptom in both diseases, but their incidence was significantly higher in the NMOSD group compared with the MS group (77.17% vs. 55.17%, *p* = 0.035), consistent with previous research findings (56). Among these, central pain is particularly prominent in NMOSD, with a significantly higher proportion presenting as an initial symptom compared with MS (45.74% vs. 17.24%, *p* = 0.006). Its pathological basis is largely attributed to extensive spinal cord lesions, which may be associated with loss of thermal sensation, central neuropathic pain, and even the modulation of pain by endogenous cannabinoids released from spinal astrocytes (53). In addition to sensory symptoms, spinal cord involvement can also lead to motor dysfunction and bladder or bowel disturbances. In this study, the incidence of these two initial symptoms in the NMOSD group (motor dysfunction 62.77%, bladder/bowel disturbances 46.81%) was significantly higher than in the MS group (41.37, 10.34%). This is closely related to the greater propensity of NMOSD to develop longitudinally extensive transverse myelitis (LETM), with lesions predominantly affecting the cervical and thoracic spinal cord and exhibiting a more extensive distribution (57, 58). Accordingly, the proportion of NMOSD patients presenting with severe muscle weakness (grade 0–3) at admission was much higher than that of MS patients (42.55% vs. 13.79%), further confirming that neurological deficits are more pronounced and disability is greater at disease onset in NMOSD. These findings underscore the importance of comprehensive symptom assessment and neurological examination in patients with acute-onset spinal cord syndromes for the early differentiation of NMOSD and MS in clinical practice.

Optic neuritis (ON) is a core symptom of NMOSD and a common manifestation of MS. ON associated with NMOSD is often more severe, prone to causing permanent vision loss, and typically involves bilateral optic nerve chiasm and optic tracts (59–61). In terms of MRI findings, NMOSD typically presents with longitudinally extensive lesions, bilateral optic nerve involvement, and posterior pathway damage, whereas MS more commonly exhibits focal lesions (62). In this study, 44.68% of NMOSD patients presented initially with visual disturbances, significantly higher than in MS patients (24.13%, *p* = 0.048). Due to the diverse range of symptoms, patients first sought care in a variety of clinical departments. Although most patients were initially seen in neurology (NMOSD 34.04%, MS 51.72%), a considerable proportion of NMOSD patients first visited ophthalmology (21.28%), gastroenterology (8.51%), and other departments, highlighting the need for enhanced multidisciplinary collaboration and diagnostic differentiation.

The differences in the aforementioned clinical manifestations were further objectively confirmed through laboratory and imaging examinations. Laboratory tests play an important role in differential diagnosis, with NMOSD patients often exhibiting elevated cerebrospinal fluid (CSF) white blood cell counts and protein levels, reflecting a more pronounced inflammatory response (63, 64). In this study, the NMOSD group exhibited higher cerebrospinal fluid (CSF) white blood cell counts, lymphocyte proportions, and protein levels compared with the MS group, likely reflecting more severe spinal cord lesions and associated inflammatory responses in NMOSD. As a specific biomarker for NMOSD, the detection of AQP4 antibodies has significantly improved diagnostic accuracy; in this study, the positivity rate was 76.19%, markedly higher than in MS patients (11.53%, *p* < 0.001) (65). The pathogenic core of NMOSD is the immune cascade triggered by AQP4-IgG. This antibody not only induces astrocyte damage but also promotes inflammatory cell infiltration and disruption of the blood–brain barrier, which in turn facilitates the entry of more antibodies into the central nervous system, creating a vicious cycle that ultimately leads to demyelination and neurological dysfunction (66). MS is more commonly characterized by positive oligoclonal bands (OCB), with a positivity rate of 65.38% in this study compared with 13.10% in NMOSD (*p* < 0.001), providing a useful tool for differential diagnosis.

Compared with laboratory indicators, neuroimaging provides a more direct basis for differential diagnosis, with MRI serving as an important tool for distinguishing between the two diseases. In MS, intracranial lesions are predominantly located around the lateral ventricles, subcortical regions, brainstem, and cerebellum, typically appearing as round or oval-shaped lesions (67). NMOSD was previously thought to involve only the optic nerves and spinal cord; however, subsequent studies have found that many cases also exhibit intracranial lesions, predominantly in regions with high AQP4 expression, such as periventricular areas, the thalamus, and the brainstem (68). The area postrema of the medulla, due to its high blood–brain barrier permeability, is a characteristic site of involvement in NMOSD (69). In this study, the rate of abnormal cranial MRI findings was higher in MS than in NMOSD (96.55% vs. 55.32%), with significant differences observed in subcortical, centrum semiovale, and periventricular lesions (*p* < 0.05). On the other hand, the hallmark spinal cord injury in NMOSD, longitudinally extensive transverse myelitis (LETM), refers to lesions spanning three or more vertebral segments. Its occurrence is related to preferential involvement of the central gray matter, where AQP4 is highly expressed. On MRI, LETM appears as lesions affecting more than 50% of the spinal cord cross-sectional area, commonly in the cervical or thoracic segments, and can result in severe sensory and motor deficits (6, 70–73). In contrast, MS more commonly presents with short-segment lesions (short segment myelitis, SSM) located in the white matter, typically distributed dorsally or laterally (74). In this study, the rate of abnormal spinal MRI findings was significantly higher in NMOSD than in MS (88.30% vs. 58.62%, *p* < 0.001), with significant differences observed in cervical, thoracic, cervicothoracic, and long-segment (≥3 vertebral segments) involvement. These findings are consistent with the more destructive clinical spinal manifestations seen in NMOSD.

Evoked potentials are useful for detecting subclinical lesions. In this study, the rate of abnormal visual evoked potentials (VEP) was higher in the NMOSD group than in the MS group (53.09% vs. 29.17%, *p* = 0.039), and NMOSD more frequently exhibited waveform absence, indicating more severe optic nerve damage (75, 76). Optical coherence tomography (OCT) can serve as a tool for assessing retinal structural damage, with NMOSD often showing more pronounced thinning of the nerve fiber layer and microcystic macular edema (77, 78). OCT examinations were not conducted in this study, highlighting the need for enhanced assessment in this area in future research.

In terms of treatment and prognosis, both NMOSD and MS in the acute phase are primarily managed with high-dose corticosteroid therapy, plasma exchange, and intravenous immunoglobulin (IVIG), aiming to alleviate symptoms, shorten disease duration, and improve outcomes (79, 80). This study showed that EDSS scores during the acute phase and 1 year after discharge were higher in NMOSD than in MS, with a significant difference observed during the acute phase (*p* < 0.05), indicating more severe attacks and poorer prognosis in NMOSD, consistent with previous research findings (81). Both diseases are characterized by high relapse and disability rates, requiring long-term immunosuppressive therapy. However, in this study, there was no significant difference between the groups in the use of immunosuppressants during remission, and overall usage was low, possibly due to economic factors or patient preference. This underscores the need for more effective and accessible treatment options to improve long-term patient outcomes.

This study has several limitations. First, as a retrospective study, some patients’ acute-phase clinical data were incomplete. Second, some patients had a long disease course, and considering patient compliance, follow-up data may be subject to bias. Third, the overall sample size was relatively small, and the imbalance between the NMOSD and MS groups may affect the reliability of the statistical results. In future research, we plan to expand the sample size and conduct large-scale, multicenter studies.

## 6 Conclusion

This study provides clinical, serological, and imaging data of NMOSD and MS patients in the Ningxia Hui Autonomous Region of China, identifies the distinguishing features of the two diseases within a unified framework, and helps to better understand the regional and ethnic differences in disease characteristics. In summary, this study demonstrates that, compared with MS patients, NMOSD patients have a later age of onset, a higher proportion of females, higher relapse rates, and greater disability. Radiologically, spinal cord lesions—particularly longitudinally extensive lesions—are more common in NMOSD, whereas intracranial MRI abnormalities are more frequently observed in MS. Additionally, abnormalities in visual evoked potentials are more prevalent in NMOSD. Although both diseases are characterized by high relapse and disability rates, they differ significantly in terms of demographics, clinical manifestations, laboratory findings, and neuroimaging features. Therefore, early differential diagnosis is essential to guide appropriate treatment strategies and improve long-term patient outcomes.

## Data Availability

The original contributions presented in the study are included in the article/Supplementary material, further inquiries can be directed to the corresponding author.
