# Supplementary material for: Differential diagnosis between NMOSD and MS: a retrospective study based on clinical and imaging features
Source: Front Neurol. 2026 Jan 20;17:1718736. doi: 10.3389/fneur.2026.1718736 (PMC12864107; doi:10.3389/fneur.2026.1718736)
Supplement: Supplementary file 1 [file Table_1.DOCX]

| **Appendix 1. 2015 NMOSD diagnostic criteria for adult patients** |
| --- |
| **Diagnostic criteria for NMOSD with AQP4-IgG** |
| 1. At least 1 core clinical characteristic |
| 2. Positive test for AQP4-IgG using best available detection method (cell-based assay strongly recommended) |
| 3. Exclusion of alternative diagnoses |
| **Diagnostic criteria for NMOSD without AQP4-IgG or NMOSD with unknown AQP4-IgG status** |
| 1. At least 2 core clinical characteristics occurring as a result of one or more clinical attacks  and meeting all of the following requirements |
| a. At least 1 core clinical characteristic must be optic neuritis, acute myelitis with LETM, or  area postrema syndrome |
| b. Dissemination in space (2 or more different core clinical characteristics |
| c. Fulfillment of additional MRI requirements, as applicable |
| 2. Negative tests for AQP4-IgG using best available detection method, or testing unavailable |
| 3. Exclusion of alternative diagnoses |
| **Core clinical characteristics** |
| 1. Optic neuritis |
| 2. Acute myelitis |
| 3. Area postrema syndrome: episode of otherwise unexplained hiccups or nausea and vomiting |
| 4. Acute brainstem syndrome |
| 5. Symptomatic narcolepsy or acute diencephalic clinical syndrome with NMOSD-typical  diencephalic MRI lesions (figure 3) |
| 6. Symptomatic cerebral syndrome with NMOSD-typical brain lesions (figure 3) |
| **Additional MRI requirements for NMOSD without AQP4-IgG and NMOSD with unknown AQP4-IgG status** |
| 1. Acute optic neuritis: requires brain MRI showing (a) normal findings or only nonspecific whitematter lesions, OR (b) optic nerve MRI with T2-hyperintense lesion or T1-weighted gadoliniumenhancing lesion extending over ＞1/2 optic nerve length or involving optic chiasm (figure 1) |
| 2. Acute myelitis: requires associated intramedullary MRI lesion extending over ≥contiguous segments (LETM) OR ≥contiguous segments of focal spinal cord atrophy in patients with history compatible with acute myelitis (figure 1) |
| 3. Area postrema syndrome: requires associated dorsal medulla/area postrema lesions (figure 2) |
| 4. Acute brainstem syndrome: requires associated periependymal brainstem lesions (figure 2) |

| **Appendix 2. The 2017 McDonald criteria for diagnosis of multiple sclerosis in patients with an attack at onset** | | |
| --- | --- | --- |
|  | **Number of lesions with objective clinical evidence** | **Additional data needed for a diagnosis of multiple sclerosis** |
| ≥2 clinical attacks | ≥2 | None* |
| ≥2 clinical attacks | 1 (as well as clear-cut historical evidence of a previous attack involving a lesion in a distinct anatomical location†) | None* |
| ≥2 clinical attacks | 1 | Dissemination in space demonstrated by an additional clinical attack implicating a different CNS site or by MRI‡ |
| 1 clinical attack | ≥2 | Dissemination in time demonstrated by an additional clinical attack or by MRI§ OR demonstration of CSF-specific oligoclonal bands¶ |
| 1 clinical attack | 1 | Dissemination in space demonstrated by an additional clinical attack implicating a different CNS site or by MRI‡ AND Dissemination in time demonstrated by an additional clinical attack or by MRI§ OR demonstration of CSF-specific oligoclonal bands¶ |
| If the 2017 McDonald Criteria are fulfilled and there is no better explanation for the clinical presentation, the diagnosis is multiple sclerosis. If multiple sclerosis is suspected by virtue of a clinically isolated syndrome but the 2017 McDonald Criteria are not completely met, the diagnosis is possible multiple sclerosis. If another diagnosis arises during the evaluation that better explains the clinical presentation, the diagnosis is not multiple sclerosis. An attack is defined in panel 1. *No additional tests are required to demonstrate dissemination in space and time. However, unless MRI is not possible, brain MRI should be obtained in all patients in whom the diagnosis of multiple sclerosis is being considered. In addition, spinal cord MRI or CSF examination should be considered in patients with insufficient clinical and MRI evidence supporting multiple sclerosis, with a presentation other than a typical clinically isolated syndrome, or with atypical features. If imaging or other tests (eg, CSF) are undertaken and are negative, caution needs to be taken before making a diagnosis of multiple sclerosis, and alternative diagnoses should be considered. †Clinical diagnosis based on objective clinical findings for two attacks is most secure. Reasonable historical evidence for one past attack, in the absence of documented objective neurological findings, can include historical events with symptoms and evolution characteristic for a previous inflammatory demyelinating attack; at least one attack, however, must be supported by objective findings. In the absence of residual objective evidence, caution is needed. ‡The MRI criteria for dissemination in space are described in panel 5. §The MRI criteria for dissemination in time are described in panel 5. ¶The presence of CSF-specific oligoclonal bands does not demonstrate dissemination in time per se but can substitute for the requirement for demonstration of this measure. | | |

| **Appendix 3. Expanded Disability Status Scale (EDSS)** | |
| --- | --- |
| Score | Content |
| 0 | Normal neurologic exam (all grade 0 in Functional Systems [FS]; Cerebral grade 1 acceptable) |
| 1.0 | No disability, minimal signs in one FS (ie, grade 1 excluding Cerebral grade 1) |
| 1.5 | No disability minimal signs in more than one FS (more than one grade 1 excluding Cerebral grade 1) |
| 2.0 | Minimal disability in one FS (one FS grade 2, others 0 or 1) |
| 2.5 | Minimal disability in two FS (two FS grade 2, others 0 or 1) |
| 3.0 | Moderate disability in one FS (one FS grade 3, others 0 or l), or mild disability in three or four FS (three/four FS grade 2, others 0 or 1) though fully ambulatory. |
| 3.5 | Fully ambulatory but with moderate disability in one FS (one grade 3) and one or two FS grade 2; or two FS grade 3; or five FS grade 2 (others 0 or 1) |
| 4.0 | Fully ambulatory without aid, self-sufficient, up and about some 12 hours a day despite relatively severe disability consisting of one FS grade 4 (others 0 or l), or combinations of lesser grades exceeding limits of previous steps. Able to walk without aid or rest some 500 meters |
| 4.5 | Fully ambulatory without aid, up and about much of the day, able to work a full day, may otherwise have some limitation of full activity or require minimal assistance; characterized by relatively severe disability, usually consisting of one FS grade 4 (others 0 or 1) or combinations of lesser grades exceeding limits of previous steps. Able to walk without aid or rest for some 300 meters |
| 5.0 | Ambulatory without aid or rest for about 200 meters; disability severe enough to impair full daily activities (eg, to work full day without special provisions). (Usual FS equivalents are one grade 5 alone, others 0 or 1; or combinations of lesser grades usually exceeding specifications for step 4.0.) |
| 5.5 | Ambulatory without aid or rest for about 100 meters; disability severe enough to preclude full daily activities. (Usual FS equivalents are one grade 5 alone, others 0 or 1; or combinations of lesser grades usually exceeding those for step 4.0.) |
| 6.0 | Intermittent or unilateral constant assistance (cane, crutch, or brace) required to walk about 100 meters with or without resting. (Usual FS equivalents are combinations with more than two FS grade 3+.) |
| 6.5 | Constant bilateral assistance (canes, crutches, or braces) required to walk about 20 meters without resting. (Usual FS equivalents are combinations with more than two FS grade 3+.) |
| 7.0 | Unable to walk beyond about 5 meters even with aid, essentially restricted to wheelchair; wheels self in standard wheelchair and transfers alone; up and about in w/c some 12 hours a day. (Usual FS equivalents are combinations with more than one FS grade 4 + ; very rarely, pyramidal grade 5 alone.) |
| 7.5 | Unable to take more than a few steps; restricted to wheelchair; may need aid in transfer; wheels self but cannot carry on in standard wheelchair a full day; may require motorized wheelchair. (Usual FS equivalents are combinations with more than one FS grade 4+ .) |
| 8.0 | Essentially restricted to bed or chair or perambulated in wheelchair, but may be out of bed itself much of the day; retains many self-care functions; generally has effective use of arms. (Usual FS equivalents are combinations, generally grade 4 + in several systems.) |
| 8.5 | Essentially restricted to bed much of the day; has some effective use of arm(s); retains some self-care functions. (Usual FS equivalents are combinations, generally 4 + in several systems.) |
| 9.0 | Helpless bed patient; can communicate and eat. (Usual FS equivalents are combinations, mostly grade 4 + .) |
| 9.5 | Totally helpless bed patient; unable to communicate effectively or eat/swallow. (Usual FS equivalents are combinations, almost all grade 4 + .) |
| 10.0 | Death due to multiple sclerosis |
